# Aspects épidémiologiques et cliniques de la tuberculose en milieu hospitalier à Bangui

**DOI:** 10.11604/pamj.2019.33.31.13442

**Published:** 2019-05-15

**Authors:** Gaspard Tékpa, Valentin Fikouma, Régis Martial Marada Téngothi, Jean de Dieu Longo, Avilah Phrygie Amakadé Woyengba, Boniface Koffi

**Affiliations:** 1 Service des Maladies Infectieuses, Hôpital de l’Amitié, Bangui, République Centrafricaine; 2 Centre de Traitement Ambulatoire de l’Infection à VIH, Hôpital Communautaire, Bangui, République Centrafricaine; 3 Faculté des Sciences de la Santé, Université de Bangui, Bangui, République Centrafricaine

**Keywords:** Tuberculose, VIH, épidémiologie, clinique, mortalité, Bangui, Tuberculosis, HIV, epidemiology, clinical, mortality, Bangui

## Abstract

La tuberculose (TB) est endémique en République centrafricaine (RCA); son taux d’incidence est estimé à 391 pour 100.000 habitants en 2015. Notre objectif était d’étudier les aspects épidémiologiques et cliniques actuels de la tuberculose en milieu hospitalier centrafricain. Il s’agissait d’une étude analytique rétrospective portant sur des patients hospitalisés dans les Services de Médecine de l’Hôpital de l’Amitié du 15 avril 2010 au 14 octobre 2011. Les données ont été collectées à l’aide d’un questionnaire puis analysées avec le logiciel Epi Info 3.5.3. Pour comparer les proportions, le test de chi-carré a été utilisé avec un seuil de significativité de 5%. Sur 220 patients inclus il y avait 128 femmes (58,18%). La moyenne d’âge était de 35,69 ± 10,65 ans. Dans 42,70% des cas, les patients étaient sans profession. La prévalence hospitalière de la TB était de 10,99%. En moyenne, 12 cas de TB étaient enregistrés chaque mois. Les signes cliniques les plus fréquents étaient: la toux chronique (71,81%), la fièvre (96,82%), l’altération de l’état général (91,36%) et le syndrome de condensation pulmonaire (63,64%). Les pathologies les plus souvent associées étaient le VIH/sida (73,36%), le paludisme (48,63%) et l’anémie (31,81%). Le délai moyen de diagnostic était de 37,65 jours. Nous avons enregistré un taux de mortalité de 18,63%. La co-infection TB/VIH et la localisation neuro-méningée de la TB étaient associées à une forte mortalité (p < 0,05). La tuberculose est une maladie fréquente à Bangui et souvent associée à l’infection à VIH. Le pronostic est sévère en cas de localisation neuro-méningée. La prévention et le contrôle de l’infection à VIH pourraient contribuer à réduire l’ampleur et de la gravité de la TB.

## Abstract (en)

Tuberculosis (TB) is endemic in the Central African Republic (CAR) with an incidence rate of 391 per 100,000 population in 2015. This study aims to analyze current epidemiological and clinical features of TB at the Hôpital de l’Amitié in the Central African Republic. We conducted an analytic retrospective study of patients hospitalized in the Department of Medicine at the Hôpital de l’Amitié from 15 April 2010 to 14 October 2011. Data were collected using a questionnaire and then analyzed with Epi info software 3.5.3. Chi-square test was used to compare proportions, using a threshold significance level of 5%. The study included 220 patients, of whom 128 were women (58.18%). The average age of patients was 35.69± 10.65 years. In 42.70% of cases, patients had no professional activity. Prevalence of tuberculosis in hospital was 10.99%. On average, 12 cases of TB were recorded each month. Most common clinical signs included: chronic cough (71.81%), fever (96.82%), alteration of the general state (91.36%) and pulmonary condensation syndrome (63.64%). The diseases most commonly associated with tuberculosis were HIV/AIDS (73.36%), malaria (48.63%) and anemia (31.81%). The mean time between symptom onset and diagnosis was 37.65 days. Mortality rate was 18.63%. TB/HIV co-infection and neuromeningeal TB were associated with a high mortality rate (p < 0.05). Tuberculosis is a common disease in Bangui and it is often associated with HIV infection. Prognosis is poor in the case of neuromeningeal involvement. Prevention and routine monitoring in HIV infected patients may contribute to reduce the extent and severity of TB.

## Introduction

La tuberculose (TB) est une maladie infectieuse fréquente et mortelle en dépit de l’existence d’un traitement efficace. Elle représente aujourd’hui à l’échelle mondiale un problème majeur de santé publique. En 2015, 10,4 millions de nouveaux de cas de TB ont été enregistrés dans le monde dont 1,2 million (11%) de co-infection avec le virus de l’immunodéficience humaine (VIH). Durant la même année, la mortalité liée à la TB a été évaluée à 1,4 millions. L’infection par le VIH a accru le poids de la tuberculose, surtout dans les populations où la prévalence de cette dernière est forte, principalement en Afrique sub-saharienne et en Asie du sud-est [1]. Dans les pays en développement, le diagnostic de tuberculose pulmonaire repose essentiellement sur l’isolement de bacilles acido-alcoolo-résistant (BAAR) à l’examen direct des expectorations. Le diagnostic des tuberculoses extra-pulmonaires est moins aisé dépendant, de la difficulté d’obtention de matériel par geste invasif (biopsie osseuse, liquide céphalorachidien, biopsie hépatique, etc.) et d’une documentation bactériologique parfois difficile (inoculum bactérien moindre) [2]. L’immunodépression induite par le VIH est un facteur modifiant les tableaux clinique et para-clinique de la tuberculose. Ainsi, de plus en plus, chez les personnes vivant avec le VIH (PVVIH), présentant des formes de tuberculose à frottis négatif et des cas de tuberculose extra-pulmonaire sont régulièrement enregistrés [3]. Le diagnostic de ces formes est souvent difficile et tardif surtout quand les ressources sont limitées ce qui est à l’origine d’une mortalité élevée [4]. En République centrafricaine (RCA), la TB est endémique, mais ses caractéristiques spécifiques en milieu hospitalier ne sont pas bien documentées. Cette étude a été conduite afin de disposer des données sur les aspects épidémiologiques, cliniques et évolutifs de la TB en vue d’améliorer la prise en charge de la maladie. L’objectif général était de décrire la tuberculose et d’identifier les facteurs en milieu hospitalier à Bangui.

## Méthodes

Il s’agissait d’une étude analytique rétrospective, réalisée à l’hôpital de l’Amitié à Bangui entre le 15 avril 2010 et le 14 octobre 2010. La population d’étude était constituée de tous les patients hospitalisés dans les trois Services de Médecine de l’Hôpital de l’Amitié (hépato-gastro-entérologie, neurologie et maladies infectieuses) durant la période d’étude. Nous avons inclus dans l’étude, les patients âgés d’au moins quinze ans chez qui le diagnostic d’une tuberculose évolutive était posé sur des arguments cliniques et para cliniques et dont le dossier médical contenaient des renseignements sur les données cliniques, paracliniques et sur les pathologies associées. Pour chaque patient, nous avons recueilli, les données épidémiologiques (âge, sexe, profession, statut matrimonial et lieu de résidence); cliniques (antécédents, manifestations cliniques et les formes cliniques de la tuberculose) et l’évolution à court terme (en fin d’hospitalisation). Les principaux critères de jugement de l’évolution à court terme étaient la régression complète de la fièvre et le gain pondéral. Le recueil des données a été fait à partir du registre d’hospitalisation et des dossiers médicaux. La fiche d’enquête était anonyme et confidentielle. Les données recueillies ont été saisies et analysées à l’aide du logiciel Epi Info 2000, version 3.5.3. Le test de chi carré ou celui de Fischer exact était utilisé pour la comparaison des proportions avec un seuil de significativité de 5%.

## Résultats

### Données épidémiologiques

**Figure 1:**
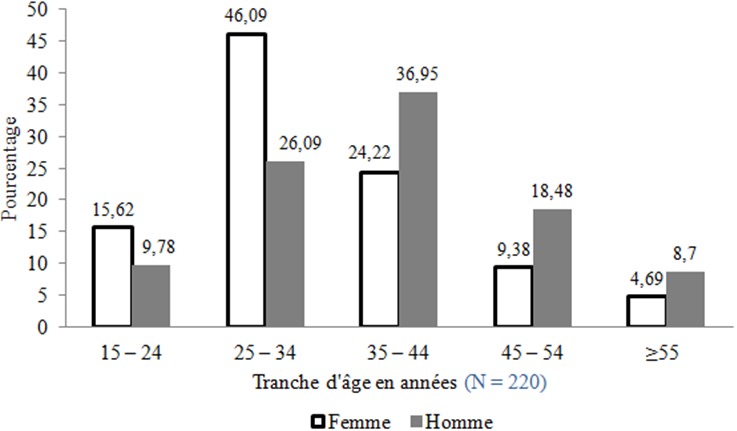
Répartition des patients atteints de tuberculose par tranche d’âge et par sexe

**Figure 2:**
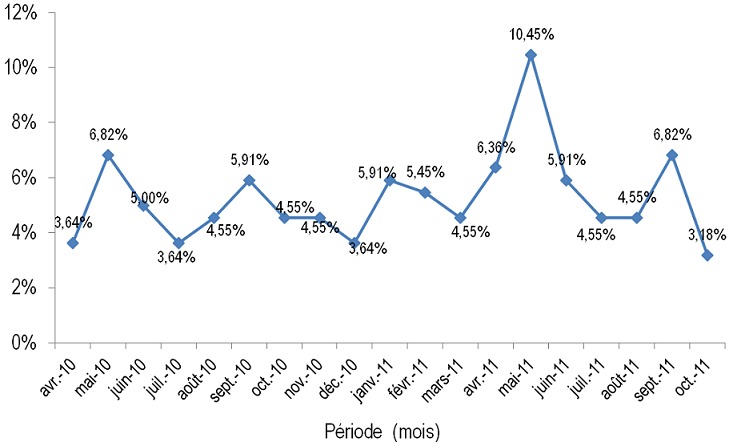
évolution de la fréquence mensuelle des cas de tuberculose survenus à l’Hôpital de l’Amitié, à Bangui entre avril 2010 et octobre 2011 (N = 220)

Au total 220 patients ont été inclus dont 128 femmes (58,18%) soit un sex-ratio de 0,71. L’âge moyen des patients était de 35,69 ± 10,65 ans; la médiane était de 34 ans avec des extrêmes de 16 et 75 ans (Figure 1). Parmi eux, 120 (54,54%) étaient des célibataires, 87 (39,55%) des mariés, 9 (4,10%), des veufs et 4 (1,81%) des divorcés. Concernant le statut professionnel, il y avait 57 salariés (25,90%) et 29 commerçants (13,2%). Les personnes sans emploi (y compris ménagère, élèves/étudiants) représentaient 51,81%. Durant la période de l’étude, 2002 malades ont été admis en hospitalisation dont 220 pour une tuberculose soit une prévalence hospitalière de 10,99%. Cette prévalence était de 27,95% (173/619), 3,92% (39/994) et 2,06% (8/389) respectivement dans les services de maladies infectieuses, d’hépato-gastro-entérologie et de neurologie. Les cas de tuberculose étaient survenus de manière continue sur toute la période de l’étude. Trois pics ont été observés respectivement en mai 2010 (6,82%), en mai 2011 (10,45%) et septembre 2011 (6,82%) (Figure 2). Le nombre moyen mensuel de cas enregistrés était de 12,22. Nous avons enregistrés 41 décès soit un taux de létalité de 18,63%. La fréquence mensuelle des décès a varié de 0 à 12,20% avec deux pics, l’un en mai 2011 (12,20%) et l’autre en septembre 2011 (12,20%). La moyenne était de 2,27 décès par mois. Dans les 10 cas où les circonstances de décès étaient précisées, le décès était secondaire à un tableau de collapsus cardiovasculaire dans 12,20%. Le délai moyen de survenu de décès était de 15,65 jours; la médiane était de 11 jours avec des extrêmes de 1 et 78 jours. Dans 75% des cas, le décès intervenait avant le 18ème jour d’hospitalisation. La tuberculose survenait sur terrain immunodéprimé à VIH, sur terrain éthylique, tabagique, diabétique et cirrhotique respectivement dans 73%, 7,73%, 3,18%, 2,27% et 0,90%. Nous avons observé un cas de tuberculose chez un asthmatique (0,45%). L’antécédent pathologique le plus fréquent était la tuberculose (8,64%). Sur 19 patients ayant signalé un antécédent de tuberculose, nous avons noté deux cas d’interruption de traitement antituberculeux et 17 cas de traitement complet soit un taux de rechute de 7,72% (17/220).

### Données cliniques et paracliniques

Les principaux signes fonctionnels enregistrés ainsi que les signes généraux ont été décrits au Tableau 1. Les autres signes fonctionnels étaient représentés par la cervicalgie, la convulsion, le vomissement, l’épigastralgie, l’hématémèse, la tuméfaction latéro-cervicale et la pollakiurie. À l’examen physique, la manifestation la plus fréquente a été le syndrome de condensation pulmonaire observé dans 63,64% des cas (Tableau 2). Le diagnostic biologique était basé sur l’examen de différents prélèvements. La recherche des bacilles acido-alcoolo- résistants (BAAR) a été positive dans les crachats chez 48 patients sur 90 patients (53,33%) ou les sécrétions obtenues par tubage gastrique deux fois sur neuf (22,22%) ou encore dans les urines chez un malade. Le recours à l’examen du suc ganglionnaire a mis en évidence des signes cytologiques caractéristiques de la tuberculose chez cinq patients sur six (83,83%). Le liquide pleural, le liquide d’ascite et le liquide cérébrospinal ont été examinés respectivement chez 38 (17,37%), 18 (8,18%) et 11 (5%) patients. L’examen des liquides de ponction (liquide pleural, liquide d’ascite, liquide cérébrospinal) était pathologique dans tous les cas et a permis de fournir des éléments d’orientation diagnostique sans mettre en évidence des BAAR. Le liquide pleural et le liquide d’ascite étaient caractérisés par un exsudat (Rivalta positif) et une élévation des leucocytes avec prédominance des lymphocytes. Dans le liquide cérébrospinal, il y avait une protéinorachie et une leucocytose à prédominance lymphocytaire. Au plan de l’imagerie médicale, sur 160 radiographies du thorax réalisées à but diagnostic, il n’y avait pas d’anomalies dans 18 cas (11,25%). Dans les 152 autres cas (88,75%), un total de 292 anomalies radiologiques ont été observées dont les plus fréquentes étaient les opacités macro-nodulaires (27,21%), les opacités micronodulaires (25,46%) et les opacités homogènes à tonalité hydrique en rapport avec une pleurésie (21,94%). L’échographie abdominale a été réalisée chez 134 patients et a permis de mettre en évidence les adénopathies profondes, l’hépatomégalie, l’ascite, la splénomégalie et des nodules spléniques respectivement dans 60 (44,77%), 47 (35,07%), 35 (26,11%), 32 (23,88%) et 12 cas (8,95%).

**Tableau 1:** Répartition des signes fonctionnels et généraux au cours de la tuberculose à Bangui selon la fréquence (n = 220)

Signes fonctionnels	Effectif	Pourcentage (%)
Toux chronique	158	71,81
Douleur thoracique	127	57,73
Douleur abdominale	88	40,00
Dyspnée	41	18,64
Diarrhée	22	10,00
Flatulence	13	5,91
Céphalée	12	5,45
Hémoptysie	11	5,00
Constipation	8	3,64
Autres	17	7,73
**Signes généraux**		
Fièvre	213	96,82
Amaigrissement	201	91,36
Asthénie	194	88,18
Anorexie	99	45,00
Sueur nocturne	45	20,45

**Tableau 2:** répartition des signes physiques observés au cours de la tuberculose à Bangui selon la fréquence (N = 220)

Signes physiques	Nombre	Pourcentage (%)
Syndrome de condensation pulmonaire	140	63,64
Pâleur conjonctivale	83	37,72
Pleurésie	50	22,73
Hépatomégalie	47	21,36
Adénopathies périphériques	44	20,00
Splénomégalie	32	14,55
Irritation péritonéale	31	14,09
Ascite	24	10,91
Œdème des membres inférieurs	20	9,09
Assourdissement des bruits du cœur	20	9,09
Syndrome méningé	8	3,64
Paraplégie	6	2,73
Ictère	4	1,82
Altération de la conscience	4	1,82
Autres	14	6,36

### Formes cliniques de tuberculose

**Figure 3:**
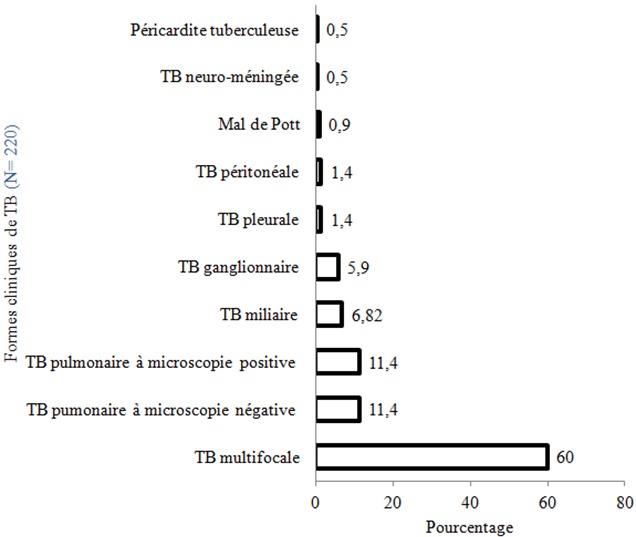
Formes cliniques de tuberculoses enregistrées à l’hôpital de l’Amitié de Bangui entre avril 2010 et octobre 2011 (N = 220)

Les principales formes cliniques de TB observées (Tableau 3) étaient la TB extra-pulmonaire 163 cas (74,09%), la TB pulmonaire 138 cas soit 62,72% (dont 80 cas (36,36%) de TB pulmonaire diagnostiquée cliniquement, 58 cas (26,36%) de TB pulmonaire bactériologiquement confirmée), la TB multifocale 132 cas (60%) et la TB miliaire 15 cas (6,81%). La localisation ganglionnaire était observée dans 34,55% (Figure 3).

**Tableau 3:** répartition des cas de tuberculose extra pulmonaire diagnostiqués à Bangui selon les sites (n = 163)

Tuberculoses extra-pulmonaires	Nombre	Pourcentage
TB ganglionnaire	76	34,55
TB pleurale	64	29,09
TB péritonéale	34	15,45
TB hépatosplénique	23	10,45
TB hépatique	20	9,09
TB splénique	11	5,00
TB neuro-méningée	8	3,64
Polyscérite tuberculeuse	6	2,73
Péricardite tuberculeuse	4	1,82
Mal de Pott	3	1,36

### Délai de diagnostic et pathologies associées

Le délai de diagnostic était connu chez 197 patients. Ce délai était inférieur à 44 jours dans 75% des cas. Chez 25% des malades, le diagnostic était connu dans les 21 jours suivant le début des signes cliniques. Le délai médian de diagnostic était de 31 jours avec des extrêmes de 3 et 218 jours. La moyenne était de 37,65 jours. Le VIH/sida et le paludisme étaient les pathologies associées les plus fréquentes, respectivement dans 168 cas (86,15%) et 107 cas (48,64%), suivis de l’anémie dans 70 cas (31,82%) et de la candidose buccale ou œsophagienne dans 45 cas (26,79%) et autres (entérite, hépatite virale, œsophagite, gastrite, cirrhose décompensée, lombo-sciatique, encéphalite virale, maladie de kaposi).

### Facteurs de risque de mortalité au cours de la tuberculose

L’étude des facteurs de risque de mortalité a montré que celle-ci était associés de manière statistiquement significative à la localisation neuro-méningée de la maladie. Dans cette dernière forme clinique, la mortalité était de 50% contre 13,79% pour la TPB+ (p = 0,03). Il n’y avait de relation statistiquement significative entre les autres variables indépendantes et la mortalité, p > 0,05 (Tableau 4).

**Tableau 4:** relation entre la mortalité liée à la tuberculose, le statut sérologique VIH, le délai de diagnostic et les formes cliniques de la maladie

Paramètres	Décès				OR (IC_95_)	p
	Oui		Non			
	Nombre	%	Nombre	%		
**Statut sérologique VIH**						
VIH + (n = 168)	33	19,64	135	80,36	6,35 (0,83 - 48,55)	0,079
VIH- (n = 27)	1	3,70	26	96,30	1	
**Délai de diagnostic (en jours)**						
≤ 30 (n= 92)	19	20,65	73	79,35	1	
˃ 30 (n = 105)	22	20,95	83	79,05	0,98 (0,49 - 1,95)	0,95
**Formes cliniques de TB**						
TPB+ (n=58)	8	13,79	50	86,21	1	
TEP (n=163)	30	18,40	133	81,60	0,70 (0,30 - 1,65)	0,424
TPB- (n=80)	19	23,75	61	76,25	0,51 (0,20 - 1,27)	0,145
TB miliaire (n= 15)	4	26,67	11	73,33	0,44 (0,11 - 1,7)	0,25
Neuro-méningée (n = 8)	4	50,00	4	50,00	0,16 (0,03 - 0,77)	0,03

## Discussion

### Aspects épidémiologiques

L’enregistrement des cas de tuberculose de manière continue durant toute la période de l’étude et la prévalence hospitalière qui est de 10,99% confirment le caractère endémique de la TB. Au service des maladies infectieuses, la tuberculose est particulièrement fréquente (27,95%). Cela s’expliquerait par le profil des patients admis dans ce service qui est caractérisé par une prédominance des personnes vivant avec le VIH. La prévalence hospitalière dans notre cas est plus élevée que celle notée au cours d’une étude réalisée entre 1998 et 2002 dans un service de maladies infectieuses à Dakar [5]. Dans notre série, les patients étaient relativement jeunes (âge moyen de 35,69 ans ± 10,65 ans). Selon la littérature, la tuberculose est une maladie de l’adulte jeune. Plusieurs études réalisées dans les pays en développement révèlent une prédominance de la tuberculose chez les adultes jeunes en pleine activité socio-économique [5-8]. La tuberculose semble toucher plus précocement les femmes que les hommes. La tranche d’âge la plus touchée chez la femme était celle de 25 à 34 ans (46,09%); chez les hommes, il s’agissait des sujets de 35 à 44 ans dans 36,96%. Pour la plupart, les patients étaient de sexe féminin (128 femmes sur 220 patients soit 58,18%). Cette forte proportion des femmes serait en rapport avec la population des malades hospitalisés qui est caractérisée par une prédominance des femmes infectées par le VIH. Cependant une prédominance masculine a été décrite par la plupart des auteurs africains [9]. Par contre au cours d’une étude réalisée à Cotonou en 1997 [8] les auteurs ont enregistré un nombre égal d’hommes et de femmes donnant un sex-ratio égale à 1. Toutes les couches sociales ont été concernées par la tuberculose. Les personnes sans emploi représentaient 42,70%. La proportion des salariés était faible (15,45%). Des auteurs sénégalais ont retrouvé une proportion légèrement plus élevée de sans emploi de 47,50% [5]. Ces résultats sont en accord avec les données de la littérature selon lesquelles, la tuberculose est une maladie liée à la pauvreté [9].

L’une des complications de la tuberculose est la rechute. Elle survient même après un traitement complet bien conduit. Dans notre étude nous avons enregistré 7,72% de cas d’antécédent de tuberculose. Il était nettement inférieur à celui retrouvé à Dakar (81%) [5]. Ces rechutes seraient dues à une réactivation endogène de l’agent pathogène, favorisée probablement par l’immunodépression induite par l’infection à VIH. Concernant les signes fonctionnels, les plus fréquents étaient la toux chronique (71,81%) et la douleur thoracique dans 127 cas (57,73%). Des auteurs sénégalais ont retrouvé 90% de toux chronique avec des signes d’imprégnation tuberculeuse en 2008 [5]. Au Togo, Tidjani *et al*. ont rapporté 93,33% de toux et 27,69% de douleurs thoraciques [10]. La différence entre nos résultats et ceux des autres auteurs serait due à la méthodologie. Notre étude s’est intéressée à toutes les formes cliniques de la tuberculose; dans les formes sans atteinte pulmonaire, la toux ou la douleur thoracique peuvent être absentes. Au cours de la tuberculose, les principaux signes généraux retrouvés étaient la fièvre au long cours dans 96,82% et l’altération de l’état général (AEG) dans 75,91%. Ce sont des signes classiques, habituels de la tuberculose. Ceci s’expliquerait par le fait que nos patients consultaient très tardivement. Ces données étaient similaires à celles observées à Dakar en 2008 qui étaient caractérisées par plus de 90% d’AEG [5]. Cependant une faible fréquence des signes généraux (19,48%) a été rapportée à Lomé [10]. Ces différences seraient dues aux variations dans le profil épidémiologique ou clinique des patients. Dans notre série, les patients avaient un faible niveau socio-économique et une co-morbidité dans la majorité des cas (infection à VIH ou paludisme). Les signes physiques étaient dominés par le syndrome de condensation pulmonaire (63,64%), la pâleur conjonctivale (37,72%), l’épanchement pleural liquidien (22,73%), l’hépatomégalie (21,36%) et les adénopathies périphériques (20%). Ces signes étaient en rapport avec l’atteinte prédominante des poumons mais aussi des localisations extra-pulmonaires de la tuberculose. Ces résultats étaient comparables observés à Dakar en 2008 [5]. Les signes cliniques de la tuberculose sont variables selon la localisation de la maladie. Dans notre travail, la tuberculose était associée à une ou plusieurs autres maladies à la fois. L’infection à VIH (86,15%) était la pathologie le plus fréquemment associée. La séroprévalence de l’infection à VIH chez les tuberculeux dans notre étude était très élevée (86,15%) et se rapprochait de celle rapportée en 2008 au Service de Maladies Infectieuses de Fann à Dakar (89,80%) [5]. Cela concorde avec la littérature selon laquelle l’infection à VIH s’accompagne d’un déficit immunitaire qui favorise la survenue de nombreuses infections opportunistes dont la tuberculose [3]. La TB pulmonaire à microscopie positive ou négative était peu fréquente. La prédominance de la localisation multifocale serait liée à la forte prévalence du VIH, souvent de découverte tardive, chez les patients atteints de tuberculose dans notre série. L’immunodépression induite par le VIH favoriserait la diffusion du bacille de Koch vers différents organes autres que les poumons.

Dans notre série, il y avait une prédominance de nouveaux cas de TB (90,90%). Au moment de l’hospitalisation, la très grande majorité des malades étaient sous quadrithérapie (RHEZ), c’est-à-dire en première phase de traitement antituberculeux. Cette proportion de malades en hospitalisation à la phase d’attaque du traitement est variable selon les études; elle a été de 25,41% en 1991 à Lomé [10] et de 61,22% en 2008 à Bamako [11]. Dans l’ensemble, l’évolution à court terme de nos patients sous traitement a été favorable (81,36%) comme ce qui a été rapporté à Lomé (84,10%) [10] et mieux encore que des taux d’amélioration observés au cours du traitement de la tuberculose en 1997 à Abidjan (40%) [12] et en 2008 à Dakar (68,70%) [5]. Très peu de complications ont été observées au cours de notre étude (8,63%) par rapport à une étude réalisée à Abidjan (22,22%) [12]. Par contre, la létalité à court terme était élevée (18,63%). Sous traitement antituberculeux bien conduit, les taux de létalité rapportés par plusieurs travaux en Afrique subsaharienne sont variables, ils vont de 6,5% à 29% [10-13]; Ceci pourrait s’expliquer par le fait que nos patients consultaient souvent à un stade tardif de la maladie et que la majorité avait une co-infection par le VIH. Chez les personnes infectées par le VIH, la présentation clinique et paraclinique de la tuberculose peut être atypique rendant difficile le diagnostic de la maladie. Cette situation entraine un retard au diagnostic et aggrave le pronostic de la tuberculose [9]. De plus, le délai de diagnostic qui était longue comme dans d’autres pays africains [5, 13], variant de 16 à 45 jours pour 60,90% de nos patients pourrait expliquer en partie cette forte létalité. La létalité liée à la tuberculose en l’absence d’infection par le VIH était faible par rapport à celle observée chez les patients tuberculeux infectés par le VIH et ceci de manière statistiquement significative (p < 0,05). La neuro-méningée de la maladie était associée à une létalité plus élevée que les autres formes cliniques avec une différence statistiquement significative (p < 0,05). Une étude antérieure réalisée en milieu hospitalier à Bangui relevait une forte mortalité liée aux infections neuro-méningées [14]. Cela peut être lié à l’insuffisance du plateau technique nécessaire pour un diagnostic précis de ces infections ou à un recours tardif aux soins ou encore aux difficultés financières ne permettant pas une prise en charge efficace.

## Conclusion

La tuberculose est une maladie fréquente en milieu hospitalier centrafricain. Elle atteint principalement les adultes jeunes surtout du sexe féminin. Les localisations extra-pulmonaires sont les plus fréquentes. La coïnfection TB/VIH est courante et représente un facteur de risque important de mortalité. Cela justifie une attention particulière dans la prise en charge de la coïnfection TB/VIH comme le recommande l’OMS.

### État des connaissances actuelles sur le sujet

- La tuberculose est une maladie liée à la pauvreté;
- Elle est plus fréquente chez les hommes que chez les femmes.

### Contribution de notre étude à la connaissance

- La tuberculose a été observée dans toutes les tranches d’âge; elle atteint toutes les couches sociales quelque soient les conditions socioéconomiques;
- La maladie prédomine chez les femmes par rapport aux hommes (sex ratio H/F = 0,71) ;
- La localisation extra-pulmonaire est plus fréquente que la forme pulmonaire de la maladie.

## Conflits des intérêts

Les auteurs ne déclarent aucun conflit d'intérêts.
